# Uncovering the Imprints of Chronic Disease on Patients’ Lives and Self-Perceptions

**DOI:** 10.3390/jpm11080807

**Published:** 2021-08-18

**Authors:** Cheryl Lin, Rungting Tu, Brooke Bier, Pikuei Tu

**Affiliations:** 1 Policy and Organizational Management Program, Duke University, Durham, NC 27705, USA; c.lin@duke.edu (C.L.); brooke.bier@duke.edu (B.B.); 2 College of Management, Shenzhen University, Shenzhen 518060, China; rungting@szu.edu.cn

**Keywords:** patient-centered, personalized care, arthritis, autoimmune disease, sociopsychological factors, emotion, depression, self-identity, qualitative study, observational study

## Abstract

Rheumatoid arthritis (RA) patients face psychological hardship due to physical discomfort, disabilities, and anxieties. Previous research indicated a bidirectional relationship and patient desire for emotional support from providers. This study examined lesser-understood RA experiences across the psychological and social contexts in relation to self-perception through the patients’ expression of their struggles with these burdens. We conducted four semistructured focus groups and eleven interviews (total *n* = 31). A codebook was developed and refined through iterative transcript coding via NVivo-12. Four emerging themes were identified by inductive, thematic analysis: (1) the patients’ healthy appearances were a myth, with subthemes revealing a conflict between an inclination to hide the disease and a desire for validation, while feeling embarrassed by symptom manifestations and disappointment at withdrawal from social interactions; (2) an identity crisis due to diminished functionality, autonomy, and sense of self; (3) RA constantly occupied the mind, as its unpredictability dictated daily schedules and altered plans; and (4) the disease’s chronic nature influenced personal outlook to worry about or accept the uncertainty. Even with effective treatment, the invisibility of the disease, the fear and anticipation of flare-ups, and identity clashes caused emotional distress. The insights offer a different perspective on personalized medicine, complementing clinical treatments based on genetic or biomarker profile. For patient-centered holistic care, education is needed to prompt both patients and providers to discuss psychological issues for more customized, integrated interventions. The findings can help inform healthcare teams and families in recognizing and supporting these physical-psychological intertwined experiences, thereby ameliorating patients’ wellbeing.

## 1. Introduction

Rheumatoid arthritis (RA) is a chronic, systematic inflammatory condition characterized by persistent and progressive joint and autoimmune dysfunction. It is estimated that up to 2% of the global population and about 1.5 million Americans suffer from RA; most are women with an age of onset between 40 and 60 years old [1,2]. The etiology of RA is relatively unknown. However, the resulting prolonged inflammation typically causes joint deformation, stiffness, excessive fatigue, as well as widespread and intense pain [3]. In addition, most patients face psychological hardship due to RA-imposed functional limitations and anxieties [4,5,6].

Numerous studies have described a bidirectional relationship between RA disease activities and poor mental functioning. The progression of RA often leads to feelings of helplessness, grief, and uncertainty [6,7,8]. This psychological distress further contributes to the intensification of pain and thus creates a vicious cycle of physical and emotional suffering [9,10,11]. RA patients have consistently reported a loss of enjoyable activities, a struggle to find and adhere to effective treatment, and difficulties in actively dealing with disease manifestations, implicating a 14–62% prevalence rate of depression in the RA community [6,12,13,14,15]. Moreover, illness-related shame and loneliness perpetuate social withdrawal, and the resulting lack of social support could lead to aggravated disease outcomes [16,17,18]. Recent studies have also discussed the impacts of the disease on self-esteem and outlook; patients express the loss of autonomy and low self-efficacy in maintaining a sense of independence due to physical disabilities [4,14,19].

These findings underscore the importance of a deeper understanding of the psychological nuances of RA’s personal impacts. Previous research has explored such experiences to help plan treatments, but often focused on improving quality of life and coping strategies [14,15,20]. Other studies have deliberated how patient-centered care can complement clinical disease management for a more holistic approach [21,22]. Patient-centeredness encompasses a bio-psychosocial perspective to provide attentive, individualized care with emotional support and augmented communication for the patients’ overall wellbeing [23,24]. While emerging personalized medicine emphasizes the individuals’ distinctive genetic or biomarker profile to inform prevention and customize treatment [25,26], the patients’ mental and contextual states are also critical in determining their response to medications and health outcomes.

Few existing studies have comprehensively explored the intricacies of these experiences faced by RA sufferers on a daily or long-term basis, or investigated personalized intervention techniques addressing such emotional and social struggles. It is imperative to hear and respect patients’ voices and incorporate multidimensional therapies targeting biological and psychological pains concordantly [27,28,29]. Research in rheumatology and other medical disciplines have predominately evaluated the patients’ conditions quantitatively [30,31]. This study utilized qualitative methods to uncover and synthesize lesser-understood psychological burdens, whether from or in combination with physical encumbrances. We captured the patients’ narratives in their own words and examined the complex existence of RA in relation to the patients’ self-perceptions and outlook in personal and social contexts. By identifying and analyzing novel domains of the impact of RA on lives and individual identities, these experiences and sentiments can be better recognized and legitimized. The findings also provide insights for more effective, personalized psychological interventions and support.

## 2. Methods

We conducted four focus groups (*n* = 20) and eleven individual interviews to elicit participants’ descriptions of RA’s presence in and influence on their lives (total *n* = 31 in 15 sessions). Eligibility criteria for participating in the study were age 18 years or older, clinically diagnosed with RA, and have received treatment for RA in the previous year. Focus-group participants were recruited and screened by a market research agency to ensure a diverse sample; two experienced moderators facilitated the four groups. Individual interviewees were recruited through flyers posted at local clinics, then screened and interviewed by the research team.

A semistructured discussion guide was constructed, incorporating inputs from the literature [6,30,32,33,34,35] and experts, and was used across all sessions. The discussions explored participants’ history of diagnosis, hobbies, what a good day or bad day was like, awareness of and sentiments towards RA’s impact or associated changes, sources and specifics of their emotional state, struggles with and strategies for managing the disease, and interactions with physicians and families. Participants were also asked to self-rate the extent to which their lives were impacted by RA on a scale of one to seven, one being “no impact” and seven being “extreme impact”.

Group and individual interviews were recorded, transcribed, and de-identified. We uploaded text data into NVivo software version 12 for coding and analysis, utilizing an inductive, thematic approach [36]. A codebook was developed and refined through iterative coding by multiple researchers. Common experiences and sentiments emerging from the data were grouped together for potential theme categorization. Disagreements regarding coding and identification or naming of themes were discussed and resolved through consensus. Selective quotes were extracted to represent the essence of each theme in the participants’ own words. The research protocol was approved by Duke University Institutional Review Board. Informed consents were obtained from individual participants before each session.

## 3. Results

### 3.1. Participant Characteristics

A total of 31 people were interviewed in groups or individually. Participants were heterogenous in terms of demographics (mean age 47.39, SD = 14.38; 77.42% female; 38.7% minorities; Table 1) and disease-related characteristics (mean disease duration: 11.76 years, SD = 8.71; mean RA impact: 4.29 on a scale of 1 to 7, SD = 1.19). The occurrences and intricacies of RA’s impact were obtained via qualitative inquiries, described narratively and illustrated in a diagram below.

### 3.2. Healthy Appearance as a Myth

#### 3.2.1. Invisibility and Lack of Validation

A common struggle highlighted by participants was others’ inability to comprehend that the participants were sick. Many endured physical impediments regularly but showed no discernible signs of disease. People often did not believe that the participants were experiencing pain or take the condition seriously, making it difficult for participants to feel supported and validated.

*“It’s one of those things you can’t see it: I look fine, I look completely fine…. It’s kind of like the silent suffering…because you don’t look any different, you don’t act any different, and I kind of learned to just deal with it.”*

*(Participant #25)*

*“I’ve wondered a lot like is this all just in my head…and how much of this is real?”*

*(#24)*

Relationships were strained by their friends’ and family’s incapacity to see the symptoms and consequently, to believe the participants or show sympathy. Friendships dissolved as people took offense when the participants had to cancel plans due to flare-ups or fatigue. This further loss of socialization brought participants to feel a sense of deprivation and resentment toward the disease.

*“A lot of friends think you just don’t want to hang out with them. I can plan and whatever I want to plan but, on the day, when it’s time to do it, if I can’t do it, I can’t do it.”*

*(#2)*

*“You have to cancel a lot on people, and that’s really depressing.”*

*(#8)*

#### 3.2.2. Preference to Hide RA from Others

Although several participants were frustrated by RA’s invisibility, others reported purposefully hiding their symptoms or not mentioning they had the disease; some felt both sentiments simultaneously. Participants intended to minimize being perceived as ailing to achieve a desired sense of normalcy. They also attempted to disguise or counter (sometimes unsuccessfully) the anguish they experienced inside.

*“(Acquaintances’ response) is what I hate, they’re like, ‘Oh my God, I can’t believe that you’re so sick’… ‘You look so healthy! How?’…I feel like sometimes they think differently of me so I don’t like sharing it with people.”*

*(#24)*

*“Every semester, I’ve had a moment where an advisor is like, ‘hey, do you maybe not wanna do this semester?’ And I’m like, ‘No, I wanna do it!’…maybe more of a fear than a hope is being able to hold a full-time job and just live a normal life in that respect.”*

*(#30)*

*“Some days my husband comes home and I’m with full makeup and full jewelry everything—I’m looking great—and he’s like ‘what’s going on here? You feel really bad today, don’t you?’ and I’m like ‘yep.’”*

*(#2)*

#### 3.2.3. Embarrassment from Physical Symptoms

Adding to their predicament of preferring to hide the disease yet desiring to be understood, participants also described discomfiture with apparent physical symptoms. More prominent disease manifestations were indicative of sickness. Such visible signs could cause embarrassment and disappointment.

*“Your knees will look swollen, or your hands. And it was kind of embarrassing to go into dating because you tried to hide your twisted fingers.”*

*(#9)*

*“I want to go walking but I can’t. It’s like excruciating pain. It’s embarrassing sometimes, because I want to do things but can’t move.”*

*(#6)*

### 3.3. Identity Crisis

Participants, moreover, indicated their dismayed realization that they were no longer the same person after diagnosis. Unable to perform daily tasks or forced to relinquish what they formerly enjoyed, many struggled to maintain their self-identity and described having difficulty recognizing themselves. The common misconception that RA only occurs at old age added to the confusion for both participants and others.

*“I couldn’t hold the book, I couldn’t do anything. I just felt so helpless, useless and that’s not my mode. I’m a very independent person, I don’t like to ask anyone for help, and so having to do that was really problematic. It was even problematic—and I’m just going to be blunt—to go to the bathroom.”*

*(#27)*

*“It was hard to think of myself as chronically ill.”*

*(#26)*

*“I am way too young to be ending up with RA because I thought of it as an older person’s disorder.”*

*(#18)*

The perpetual exertion of managing or suppressing symptoms took a toll both mentally and physiologically. Many participants experienced self-doubt, frequent mood-swings, and long-term changes to their personalities or worldview.

*“I feel like I’m not as happy-go-lucky as I would like to be because it nags at you, distracts your mind.”*

*(#31)*

*“I was losing my mind. This is not like me.”*

*(#7)*

*“I was a very social person. I don’t go out anymore...except for going to the doctors. I stopped any social engagement…it affects EVERY part of life. I was living in a black-and-white world (with no color).”*

*(#21)*

RA also impacted fulfillment of responsibilities, which participants found upsetting and frustrating. They felt that their functionality as a parent, spouse, or employee was inadequate, and their performance of their roles within the household, office, or society seemed unsatisfactory. Some were confounded by the reversed role from caregiver to care-receiver.

*“The days I can’t are the days my kids want to do everything, like ‘mom I want to do this, ride bikes,’ and I’m like ‘talk to your dad.’ …that for me, is hurtful.”*

*(#5)*

*“I couldn’t have kids. And that’s really sad when you come from a Latino family, and the family is the nucleus of everything.”*

*(#9)*

*“I left a really good job. I just couldn’t handle the pressure and the stress with the RA symptoms.”*

*(#20)*

### 3.4. Occupying the Mindset

RA became the determinant of all activities and plans. Many participants reported constantly thinking about RA, and that the fluctuating severity of symptoms dictated continuously how their day went. Even on a “good day” with minimal symptoms, the continuous dread or anticipation of pain or incapacity remained a burden.

*“I think about it every morning when I put my feet on the floor. Like when I get up, I know what kind of day it’s going to be based on how I get up out of bed.”*

*(#12)*

*“I get nervous, I know I will have pain even before I can feel it.”*

*(#22)*

Participants also discussed the malevolent effects of RA on maintaining a sense of consistency in their lives and on their schedule. They regularly had to arrange for an alternative, bothersome plan. Many also shared nuisances due to the unpredictability of each day, whether simply getting out of bed, running errands, going to work, or taking a trip.

*“I would get up and leave for work earlier so that I could go and sleep in my car for an extra hour before going into the office. Even the driving, I would get fatigued…some days are just absolutely horrible.”*

*(#15)*

*“I might have a day when I’m feeling pretty good…but then there’s a day when I don’t …so then I get very frustrated because there are things I want to do or plan to do.”*

*(#17)*

*“It’s really difficult to make plans living like that. I just committed myself to a 3-day trip with several friends and I’m wondering am I going to be able to go? And if I go, am I going to end up in the hotel the whole time?”*

*(#13)*

### 3.5. Chronic Nature of the Disease

The chronic nature of RA led to fear and despair, especially in the early stages of diagnosis. Participants expressed annoyance with the unknown of the disease and worried about their future, even with effective treatment or minor symptoms.

*“It started in my forties, and so does that mean that when I’m in my sixties, is it going to be really bad? And how long can it be maintained at a reasonable level? What happens when something isn’t working and it hurts? And how bad can it be?”*

*(#14)*

*“The whole frustrating part is I have no idea what to expect moving forward.”*

*(#26)*

As participants reluctantly adapted to an altered life, many found ways to improve their conditions by modifying their exercise routines, diets, or perspectives through positive thinking. Some had reached a neutral sense of acceptance or became at peace with RA. Through their experience battling the disease, participants described a sense of personal growth, gaining control and confidence in being able to persevere.

*“I know I would have to take the medication for life, there is no cure. What can I do? It happens to me and I will just face it.”*

*(#29)*

*“I’m the kind of person that says other people have it worse. So I find someone who has it worse than me and I say I can do it, I can survive.”*

*(#27)*

**Figure 1:**
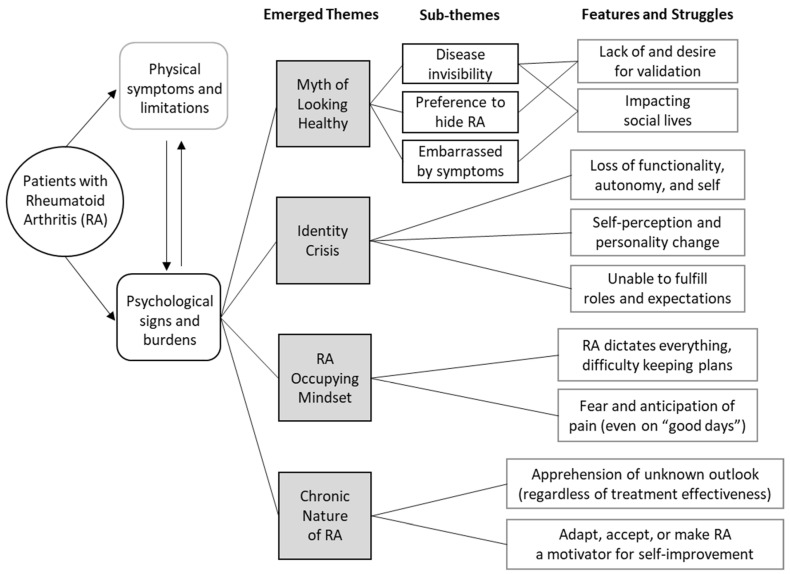
Identified themes and struggles from RA patient interviews.

We further synthesized the themes and findings in Figure 1.

## 4. Discussion

We identified four interconnected themes illustrating the imprints of RA on participants’ lives and identities through in-depth focus groups and interviews. Their narrated experiences conveyed vivid accounts of the common mental and physical dilemmas patients regularly face. Participants expressed an overarching sense of frustration, apprehension, and lack of control in both managing RA symptoms and dealing with related social and psychological consequences. However, many were able to eventually come to terms with the illness and adapt, albeit reluctantly, to their new normal.

Earlier studies examined patients’ preference for hiding symptoms and a desire for validation separately [4,37]. We uncovered the RA patients’ concurrent intentions to conceal the disease and its manifestations while still seeking recognition to escape the disease’s isolating effects. The suppression of knowledge about their illness might have made the disease feel even more confining and their experience invalidated. Other diseases have also been described as invisible, including fibromyalgia [38], chronic fatigue [39], and inflammatory bowel disease [40]. Our participants’ qualitative reflections shed light on the seemingly contradictory inclinations encountered by RA and other patient populations.

These clashing sentiments are analogous to previous findings of RA’s social impact, wherein patients described “illness-related shame” in being perceived as sickly and incapable [18]. Although the literature did not indicate whether this stigmatization was actualized or self-promulgated, our findings suggest a combination of both. Some participants were teased or treated differently by friends and family, but others tended to place guilt on themselves for not living up to expected roles. This self-disappointment was especially hurtful in a family context; such experiences were similar to that of mothers pervaded with guilt due to limited involvement in or forced exclusion from enjoyable family moments [41,42].

Moreover, our participants echoed an identity crisis pertaining to losing independence or self-esteem and feeling useless, which may contribute to personality change or depression. Participants were perceived differently by others, and came to (not) recognize themselves internally. These observations build upon existing understandings of RA-related identity conflict, where patients describe poor body-self unity in a physical sense [34,43]. Some felt uncomfortable in their own skin due to pain and bodily changes [5]. Others saw themselves as increasingly disabled and ineffective, propagating low self-confidence [7,8]. These findings highlight the far-reaching effect of RA on self-perception and altering identity. Both providers and support systems need to address these emotional hardships of RA patients, as psychological distress could lead to worsened disease and related mental health outcomes [16,44]. This is where patient-centered, integrative care involving a multidisciplinary consultation could be most beneficial [21,23].

Adding to the literature of disease-associated anxiety [6,16], our results underlined how RA remained prominent in the participants’ minds. The ever-presence of the disease amended all plans and routines, and thereby minimized any sense of consistency. The participants further disclosed sentiments ranging from anxiety and concern about their future to neutrality and acceptance of the disease being part of their lives with the unending need for maintenance. However, even with effective treatment or absence of pain, the chronic nature of RA was nevertheless frustrating and worrisome for both new and seasoned patients. Many participants not only feared and actively avoided potentially distressing circumstances, but lived in a perpetual state of anticipation of symptom manifestation and a terror of possible disability. Personalized medicine needs to account for these non-somatic factors that could influence how patients take or react to medication, disease progression, and health outcomes.

Previous research has connected behavior modifications to the disease’s conditions with positive results, such as decreased intensity of depression, reduced feelings of guilt and hopelessness, and improved physical functions [8,14,42]. Although no action may entirely remove the afflictions patients face, we also found encouraging evidence of patients turning illness impediments into motivation for self-improvement in other areas of their lives. Future exploration of this transformative change of mindset could offer valuable lessons for personalized therapies.

Analysis of patient perspectives from their own narratives is critical for supporting this population and understanding their reality. Yet the clinical practices necessary to recognize and validate these psychological symptoms and forced alterations remain sparse [20,45,46]. Instead, treatment strategies have primarily focused on the pharmacologic and physical aspects. Healthcare professionals tend not to probe for cognitive and emotional problems beyond the more frequent diagnoses of anxiety and depression [12,14]. Patients have indicated a desire for discussing social and emotional issues with their care team but less than a quarter of them receive such support [47]. Studies concur that this exclusion of psychological signs or inquiries is not a reflection of poor healthcare quality, but rather demonstrates the need for a greater understanding of the far-reaching impact of patients’ struggles and multifaceted approaches to communication and treatment techniques [4,12,48]. The results of our qualitative investigation provided deeper insights into the complexity of psychological burdens in the RA population and informed education for both patients and providers for more holistic care. Future research into personalized medicine could explore the feasibility and effect of incorporating mental support in designing and evaluating treatment programs.

Numerous techniques have been proposed to better meet the psychological needs of RA patients, including encouraging patient involvement in medical decisions [15] and creating long-term goals [28], asking open-ended questions about the psychological impacts to allow for emotional venting [12,49], cognitive behavioral therapy [50], and the use of support groups [15,51]. These recommendations can help patients better address disease uncertainty and feelings of invalidation and uselessness. However, the effectiveness of these strategies on lesser-understood struggles has not been directly studied [47,48]. In addition, research is needed to determine if and how such therapies are actively sought by patients, or if physicians and nurses must encourage psychological counseling options as a complement to medications. Educational programs should also target the public so that families and communities can adequately offer support.

The current study has several limitations. First, the sample size was small. Different from quantitative studies, it is customary for qualitative studies to have a few dozen participants. A large majority of the participants were women, despite attention being paid to specifically recruit males as well, reflective of the doubly high incidence of RA in females (male−female ratio of RA prevalence is about 1:3) [2]. Future studies could incorporate more male perspectives, as existing findings are inconsistent as to whether men experience fewer, or perhaps differently expressed, psychological symptoms [28,34,48]. The heterogeneity of our participants in their demographic and disease-related characteristics ensured that the diverse perspectives of RA patients were represented. The subjective nature of qualitative coding could have introduced biases in the selection of themes and quotes. Other key ideas may have been overlooked due to confirmation or experimenter bias. Efforts were taken to reduce biases through consultation of the literature in developing the codebook and the utilization of multiple coders. Lastly, the volunteer-based sampling, though practical and common for similar observational studies, may have excluded patients who had different opinions or experiences but were unwilling or unable to participate.

## 5. Conclusions

There is a lack of qualitative research in rheumatic diseases and healthcare in general [30,52], signaling a gap in the literature. Future research should incorporate more in-depth examinations into patients’ needs as well as investigate illness-induced cognitive and emotional processes that influence health outcomes. Our study provides a novel, comprehensive perspective of RA patients’ experiences beyond somatic functionality and across a variety of contexts. Highlighting and connecting the sociopsychological burdens resulting from physical limitations adds to the knowledge of the impact of a chronic disease. These insights demonstrate the persistent need and the patients’ desire for more personalized care that includes psychological support. This need has also been recognized in treatment guidelines but the demand has not been met, leaving care provision incomplete for many patients. In addition to providing pharmaceutical therapies, healthcare professionals must acknowledge and address these struggles when discussing and planning treatment strategies to both validate and ameliorate patients’ experiences.

## Figures and Tables

**Table 1:** Participant self-reported demographic and disease-related information.

Participant #	Gender	Race/Ethnicity	Employment Status	Household Income (USD)	Age (Years)	RA Impact *	Duration of Disease
1	Male	White	Disabled	<25 K	64	6	42 years
2	Female	Black	Unemployed	25–50 K	45	6	18 years
3	Male	White	Retired	25–50 K	67	4	4 years
4	Female	White	Retired	150–200 K	70	4	15 years
5	Female	Hispanic	Full Time	25–50 K	40	2	5 years
6	Female	Black	Full Time	50–75 K	50	3	4 years
7	Female	White	Full Time	25–50 K	47	3	19 years
8	Female	White	Part Time	100–150 K	60	4	20 years
9	Female	Hispanic	Full Time	50–75 K	38	4	10 years
10	Male	Black	Full Time	75–100 K	34	3	8 years
11	Female	Hispanic	Full Time	50–75 K	40	5	7 years
12	Male	White	Full Time	150–200 K	49	6	15 years
13	Female	White	Disabled	25–50 K	48	5	5 years
14	Male	White	Retired	100–150 K	52	5	5 years
15	Female	White	Retired	25–50 K	72	5	9 years
16	Female	White	Part Time	>200 K	41	4	10 years
17	Female	White	Part Time	75–100 K	46	6	16 years
18	Male	Black	Full Time	100–150 k	53	6	5 years
19	Female	White	Disabled	100–150 K	40	4	13 years
20	Female	White	Full Time	150–200 K	61	4	10 years
21	Female	Asian	Unemployed	100–150 K	65	3	35 years
22	Female	Asian	Full Time	25–50 K	58	4	10 years
23	Male	White	Student	150–200 K	22	4	10 years
24	Female	Hispanic	Student	25–50 K	23	4	7 years
25	Female	White	Full Time	50–75 K	28	6	13 years
26	Female	White	Full Time	50–75 K	37	4	2.5 years
27	Female	White	Unemployed	>200 K	62	2	10 years
28	Female	Asian	Unemployed	50–75 K	48	6	11 years
29	Female	Asian	Unemployed	<25 K	33	3	17 years
30	Female	White	Student	>200 K	18	4	7 years
31	Female	White	Unemployed	150–200 K	58	4	2 years

# Participant number. * Participants were asked to rate how RA has impacted their life on a scale of 1–7, from “no impact” to “extreme impact”.

## Data Availability

The portion of the de-identified transcript directly pertaining to this paper is available from the corresponding author for one year from the date of publication upon reasonable request with a methodically sound proposal.
